# Association between ambient fine particulate matter and hemorrhagic stroke in Yancheng, China

**DOI:** 10.3389/fpubh.2026.1882030

**Published:** 2026-08-31

**Authors:** Kai Qian, Xiaorong Xu, Chunxiang Xu, Yuanfeng Jiao, Qian Sun, Xiaofeng Yu, Lingzheng Sun

**Affiliations:** 1 Department of Neurology, Dongtai People's Hospital, Affiliated Dongtai Hospital of Nantong University, Yancheng, China; 2 Department of Neurology, The First People's Hospital of Yancheng, The Yancheng Clinical College of Xuzhou Medical University, Yancheng, China; 3 Department of Respiratory Medicine, The First People's Hospital of Yancheng, The Yancheng Clinical College of Xuzhou Medical University, Yancheng, China; 4 Department of Neurosurgery, The First People's Hospital of Yancheng, The Yancheng Clinical College of Xuzhou Medical University, Yancheng, China

**Keywords:** air pollution, case-crossover, hemorrhagic stroke, hospitalization, PM_2.5_

## Abstract

**Objectives:**

Ambient air pollution, particularly fine particulate matter (PM_2.5_), has been linked to cerebrovascular disease. However, findings on the short-term effects of PM_2.5_ exposure on hemorrhagic stroke (HS) have been inconsistent across studies. Therefore, this study investigated the association between short-term PM_2.5_ exposure and HS hospitalization in Yancheng, China.

**Methods:**

An individual-level time-stratified case-crossover study was conducted using hospitalization records of patients with HS from four major hospitals in Yancheng between 2022 and 2024. Conditional logistic regression models were applied to estimate the associations between short-term PM_2.5_ exposure and HS hospitalization across different lag structures. Stratified analyses were further performed according to sex, age, hypertension, diabetes, smoking, alcohol consumption, and season.

**Results:**

A total of 2,312 patients hospitalized for HS were included in the analysis. The strongest association was observed at lag01, where each 10 μg/m^3^ increase in PM_2.5_ concentration was associated with a 3.24% increase in HS hospitalization (95% CI: 0.55%−6.00%). Significant increases were also observed at lag0 and lag1, with corresponding effect estimates of 2.50% (95% CI: 0.11%−4.95%) and 2.76% (95% CI: 0.32%−5.27%), respectively. The associations remained significant after adjustment for ozone (O_3_) but were no longer statistically significant after adjustment for sulfur dioxide (SO_2_), nitrogen dioxide (NO_2_), and carbon monoxide (CO). Relatively stronger effect estimates were observed among females, older adults, individuals with hypertension or diabetes, smokers, alcohol consumers, and during the cold season.

**Conclusion:**

Short-term PM_2.5_ exposure was associated with an increased risk of HS hospitalization in Yancheng, China, with the strongest effects observed within the first two days after exposure. The associations were more pronounced among susceptible populations and during the cold season.

## 1 Introduction

Stroke is a leading cause of death and disability worldwide (1, 2). According to GBD 2021, stroke accounted for approximately 7.3 million deaths globally, with hemorrhagic stroke (HS) contributing substantially to this mortality burden (1). In China, 1.173 million new cases of HS were estimated, with an age-standardized incidence rate of 61.2 per 100,000 population (1, 3). Compared with ischemic stroke, non-traumatic HS is characterized by abrupt onset, rapid progression, higher case fatality, and more severe disability, making it a major challenge in stroke prevention and control (4, 5). Risk factor modification remains central to the prevention of HS. Established determinants, including age, sex, genetic susceptibility, hypertension, smoking, unhealthy diet, and physical inactivity, have been widely recognized. In addition to these individual-level factors, growing evidence suggests that environmental exposures may also contribute to stroke occurrence.

Among environmental exposures, outdoor air pollution, particularly fine particulate matter (PM_2.5_), has received increasing attention as a potential contributor to HS. Previous studies have suggested that PM_2.5_ exposure may induce systemic inflammation, oxidative stress, endothelial dysfunction, autonomic imbalance, blood pressure elevation, and disruption of vascular homeostasis, thereby increasing the vulnerability of cerebral vessels to rupture (6–8). In the Women's Health Initiative, long-term PM_2.5_ exposure was linked to a higher risk of cerebrovascular events, with broadly consistent associations across stroke etiologies (9). Similarly, a case-crossover study conducted in Augsburg, Germany, reported increased stroke events following short-term exposure to particulate matter and nitrogen dioxide, with positive associations mainly observed for transient ischemic attacks and HS (10). Two studies from China have also reported relevant findings: a province-wide study in eastern China found that short-term exposure to PM_2.5_ constituents was associated with increased HS mortality, while a large cohort study in Shandong reported that long-term PM_2.5_ exposure was associated with higher mortality among patients with HS (11, 12). However, effect estimates have varied across studies, possibly owing to differences in particle size fractions, exposure windows, population susceptibility, and stroke subtype definitions. These inconsistencies underscore the need for further studies focusing specifically on HS.

Therefore, we conducted an individual-level case-crossover study in Yancheng, China, to assess the association between short-term PM_2.5_ exposure and hospitalization for HS, and to identify potentially susceptible subpopulations and seasonal patterns through stratified analyses by sex, age group, and season.

## 2 Materials and methods

### 2.1 Data collection

This study was conducted in Yancheng, Jiangsu Province, China, a city located in the northern subtropical zone and characterized by a subtropical monsoon climate. Individual-level hospitalization records were obtained from the electronic medical record systems of four major hospitals serving the study area: Yancheng First People's Hospital, Yancheng Third People's Hospital, Yancheng Hospital of Traditional Chinese Medicine, and Tinghu District People's Hospital. Collectively, these hospitals provide approximately 90% of hospital services in the urban area of Yancheng and cover nearly all urban hospitalizations for HS. Cases were identified according to the International Classification of Diseases, 10th Revision (ICD-10) codes I60–I62, including subarachnoid hemorrhage, intracerebral hemorrhage, and other non-traumatic intracranial hemorrhage. For patients admitted with HS between January 1, 2022, and December 31, 2024, demographic characteristics, residential addresses, and relevant clinical information were extracted from electronic medical records. The study was approved by the Ethics Committee of Yancheng First People's Hospital (approval number: 2025-k(YJ)-155), and the requirement for informed consent was waived. All procedures complied with the ethical principles of the Declaration of Helsinki, as revised in 2013.

Following previous time-stratified case-crossover studies of ambient air pollution, fixed-site monitoring data were used to estimate population-level ambient air pollution exposure (13–16). Air pollution records were obtained from the Yancheng Environmental Monitoring Center, which operates five fixed monitoring stations in urban Yancheng. Daily concentrations of PM_2.5_, sulfur dioxide (SO_2_), nitrogen dioxide (NO_2_), carbon monoxide (CO), and ozone (O_3_) were summarized as citywide 24-h mean values by averaging measurements across available stations. For the case-crossover analysis, pollutant concentrations were linked to each patient's event day and matched control days by calendar date. Patients residing more than 10 km from the nearest monitoring station were excluded to reduce exposure misclassification. To account for the potential influence of weather conditions, daily mean temperature (Temp) and relative humidity (Rh) were obtained from the Yancheng Meteorological Bureau and matched to the same dates.

### 2.2 Statistical analysis

We applied a time-stratified case-crossover design to assess the association between short-term PM_2.5_ exposure and hospitalization for HS. This design is widely used in environmental epidemiology to examine the acute health effects of transient exposures, such as air pollution (16–18). Each patient serves as their own control, thereby reducing confounding from stable individual characteristics, such as sex, genetic background, and long-term socioeconomic conditions. Missing data were assessed before statistical analysis. No missing values were identified among the variables included in the analyses, and no imputation procedures were performed.

The date of HS hospitalization was defined as the case day. Control days were selected from the same month and year and matched by day of the week, resulting in three or four control days for each case. Within each matched set, PM_2.5_ concentrations on the case day and corresponding control days were compared using conditional logistic regression models to estimate the association between short-term exposure and HS hospitalization. To characterize lagged effects, we examined single-day lag exposures from lag 0 to lag 3, as well as moving averages over lag 0–1, lag 0–2, and lag 0–3 days. Consistent with previous studies, potential non-linear confounding by weather was controlled using natural cubic splines for meteorological variables. Specifically, a 4-day moving average of daily mean temperature was modeled with 6 degrees of freedom, and same-day relative humidity was modeled with 3 degrees of freedom.

To examine whether the association varied across potentially susceptible subgroups, we conducted stratified analyses using the lag01 exposure metric, which showed the strongest association in the primary analysis. The analyses were stratified by sex, age (< 65 and ≥65 years), hypertension, diabetes, smoking status, alcohol consumption, and season of admission. Seasons were categorized as the warm season (April–September) and the cold season (October–March of the following year). Differences in effect estimates between strata were evaluated using the Z test:


$$\begin{array}{l} Z=\frac{\beta_{1}-\beta_{2}}{\sqrt{\left(SE_{1}^{2}+SE_{2}^{2}\right)}} \end{array}$$

where β*1* and β*2* represent the estimated regression coefficients for the two subgroups, and SE_1_ and SE_2_ denote their corresponding standard errors. Effect estimates were expressed as percent changes in the risk of HS hospitalization and corresponding 95% confidence intervals (CIs) associated with each 10 μg/m^3^ increase in PM_2.5_ concentration. Percent changes were calculated from the regression coefficient using the following formulas:


$$\begin{array}{l} \text{Percent change}=\left[\exp\left(\text{β}\times 10\right)-1\right]\times 100\text{\%}\\ \text{ Lower }95\text{\% CI}=\left[\exp\left(\left(\text{β}-1.96\times \text{SE}\right)\times 10\right)-1\right]\times 100\text{\%}\\ \text{ Upper }95\text{\%CI}=\left[\exp\left(\left(\text{β}+1.96\times \text{SE}\right)\times 10\right)-1\right]\times 100\text{\%} \end{array}$$

where β is the regression coefficient and SE is its standard error.

All statistical analyses were performed using R software version 4.5.2. Conditional logistic regression models were fitted using the survival package, and natural cubic splines were constructed using the splines package. A two-sided *P* value < 0.05 was considered statistically significant.

## 3 Results

A total of 2,312 patients hospitalized for HS were included in the analysis, with 680 admissions in 2022, 772 in 2023, and 860 in 2024. The mean age was 61.83 ± 8.13 years, and 1,523 patients (65.9%) were male. Patients aged 65 years or older accounted for 47.5% of the cohort. Hypertension was the most common comorbidity, documented in 1,643 patients (71.1%), followed by smoking (12.4%), diabetes (11.8%), and alcohol consumption (8.0%). Admissions were more frequent in the cold season than in the warm season (58.6% vs. 41.4%).

The distributions of air pollutant concentrations and meteorological factors during 2022–2024 are summarized in Table 1. Daily PM_2.5_ concentrations in Yancheng showed considerable variation, ranging from 2.46 to 208.86 μg/m^3^. The mean PM_2.5_ concentration was 29.13 ± 22.68 μg/m^3^, with a median of 22.39 μg/m^3^ and an interquartile range of 13.88–38.06 μg/m^3^. The mean concentrations of SO_2_, NO_2_, O_3_, and CO were 7.05 ± 2.10 μg/m^3^, 18.76 ± 11.68 μg/m^3^, 81.72 ± 27.48 μg/m^3^, and 0.55 ± 0.18 mg/m^3^, respectively. The average daily temperature and relative humidity were 15.66 ± 9.69°C and 72.96 ± 13.30%, respectively.

**Table 1:** Daily mean concentrations of air pollutants and meteorological factors in Yancheng, China, 2022–2024.

Variables	Min	P25	Median	P75	Max	Mean	SD
PM_2.5_ (μg/m^3^)	2.46	13.88	22.39	38.06	208.86	29.13	22.68
SO_2_ (μg/m^3^)	3.62	5.67	6.58	7.95	18.55	7.05	2.10
NO_2_ (μg/m^3^)	2.76	10.27	15.56	23.67	69.49	18.76	11.68
O_3_ (μg/m^3^)	14.57	60.95	80.52	99.64	183.14	81.72	27.48
CO (mg/m^3^)	0.16	0.43	0.53	0.65	1.27	0.55	0.18
Temp (°C)	−6.21	6.73	16.13	24.19	33.67	15.66	9.69
Rh (%)	33.61	63.04	74.63	82.69	100.00	72.96	13.30

PM_2.5_, particles with aerodynamic diameter < 2.5 μm; SO_2_, sulfur dioxide; NO_2_, nitrogen dioxide; O^3^, ozone; CO, carbon monoxide; Temp, temperature; Rh, relative humidity; Min, minimum; P25, 25th percentile; P75, 75th percentile; Max, maximum; SD, standard deviation.

Spearman correlation coefficients among air pollutants and meteorological factors are presented in Table 2. PM_2.5_ showed a moderate positive correlation with SO_2_ (*r* = 0.40) and strong positive correlations with NO_2_ (*r* = 0.74) and CO (*r* = 0.74), suggesting partly shared emission sources. Conversely, PM_2.5_ was negatively correlated with temperature (*r* = −0.46) and relative humidity (*r* = −0.30), while its correlation with O_3_ was weak and not statistically significant (*r* = −0.05). Temperature was positively correlated with O_3_ (*r* = 0.55) but strongly inversely correlated with NO_2_ (*r* = −0.67).

**Table 2:** Spearman correlation coefficients for air pollutants and meteorological factors.

Variables	PM_2.5_	SO_2_	NO_2_	O_3_	CO	Temp	Rh
PM_2.5_	1.00						
SO_2_	0.40^**^	1.00					
NO_2_	0.74^**^	0.40^**^	1.00				
O_3_	−0.05	0.07^*^	−0.39^**^	1.00			
CO	0.74^**^	0.20^**^	0.58^**^	−0.10^**^	1.00		
Temp	−0.46^**^	−0.12^**^	−0.67^**^	0.55^**^	−0.28^**^	1.00	
Rh	−0.30^**^	−0.38^**^	−0.35^**^	−0.14^**^	−0.11^**^	0.30^**^	1.00

PM_2.5_, particles with aerodynamic diameter < 2.5 μm; SO_2_, sulfur dioxide; NO_2_, nitrogen dioxide; O_3_, ozone; CO, carbon monoxide; Temp, temperature; Rh, relative humidity. ^*^P values < 0.05; ^**^P values < 0.01.

In the single-pollutant model, each 10 μg/m^3^ increase in PM_2.5_ was associated with higher hospitalization for HS on the current day and the previous day, with percent changes of 2.50% (95% CI: 0.11, 4.95) at lag 0 and 2.76% (95% CI: 0.32, 5.27) at lag1 (Table 3). The largest estimate was observed for the 2-day moving average exposure at lag01, corresponding to a 3.24% increase in HS hospitalization (95% CI: 0.55, 6.00). After adjustment for SO_2_, NO_2_, and CO in two-pollutant models, the estimates were attenuated and no longer statistically significant, whereas the associations remained significant after adjustment for O_3_ at lag1 and lag01, with percent changes of 2.55% (95% CI: 0.06, 5.11) and 3.10% (95% CI: 0.32, 5.96), respectively. No significant associations were observed at longer lag periods, indicating that the increase in HS hospitalization associated with PM_2.5_ exposure was mainly concentrated within the current and previous day of exposure.

**Table 3:** Percent changes and 95% confidence intervals in hospitalization for hemorrhagic stroke associated with each 10 μg/m^3^ increase in PM_2.5_ across different lag periods in single-pollutant and two-pollutant models.

Lag days	PM_2.5_	+SO_2_	+NO_2_	+CO	+O_3_
Lag0	**2.50 (0.11, 4.95)**	2.34 (−0.47, 5.23)	2.52 (−0.60, 5.73)	1.65 (−2.29, 5.74)	2.46 (−0.01, 4.99)
Lag1	**2.76 (0.32, 5.27)**	2.73 (−0.14, 5.69)	2.88 (−0.23, 6.09)	2.72 (−1.24, 6.83)	**2.55 (0.06, 5.11)**
Lag2	0.42 (−2.07, 2.97)	0.49 (−2.32, 3.38)	0.25 (−2.78, 3.38)	0.55 (−3.24, 4.49)	0.48 (−2.05, 3.07)
Lag3	0.45 (−2.06, 3.03)	0.54 (−2.30, 3.46)	0.31 (−2.75, 3.47)	−0.02 (−3.89, 4.01)	0.42 (−2.13, 3.05)
Lag01	**3.24 (0.55, 6.00)**	3.05 (−0.17, 6.36)	3.33 (−0.21, 6.99)	2.42 (−2.01, 7.04)	**3.10 (0.32, 5.96)**
Lag02	2.92 (−0.09, 6.02)	2.55 (−0.99, 6.20)	2.78 (−1.09, 6.79)	1.61 (−3.14, 6.58)	3.00 (−0.11, 6.21)
Lag03	2.89 (−0.45, 6.34)	2.39 (−1.44, 6.36)	3.02 (−1.16, 7.37)	1.77 (−3.28, 7.08)	2.95 (−0.48, 6.50)

PM_2.5_, particles with aerodynamic diameter < 2.5 μm; SO_2_, sulfur dioxide; NO_2_, nitrogen dioxide; CO, carbon monoxide; O_3_, ozone. Lag 0, the current day; Lag 1, 1 day before admission; Lag 2, 2 days before admission; Lag 3, 3 days before admission; Lag 01, the 2-day moving average of lag 0 and lag 1; Lag 02, the 3-day moving average of lag 0 to lag 2; Lag 03, the 4-day moving average of lag 0 to lag 3. Statistically significant associations are shown in bold.

**Figure 1:**
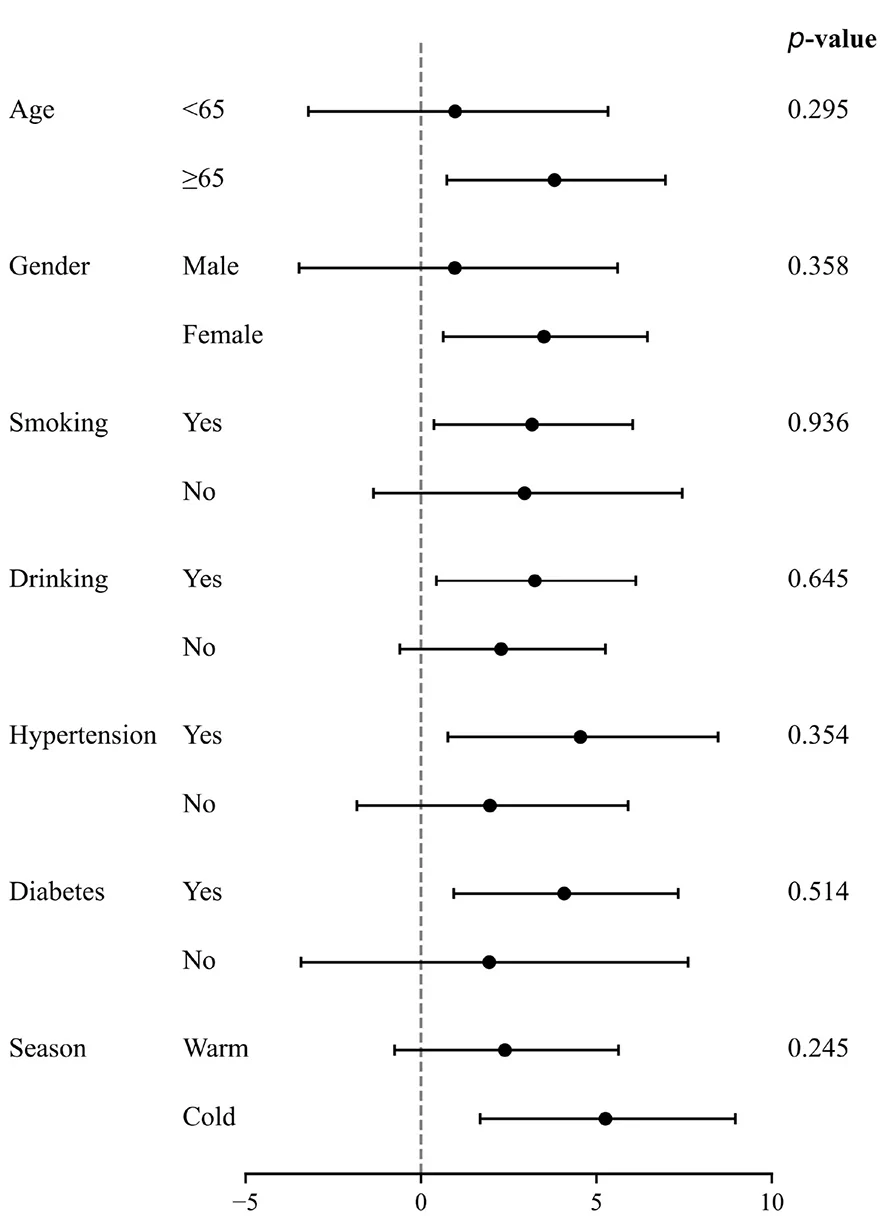
Percentage changes and 95% confidence intervals in hospitalization for hemorrhagic stroke associated with each 10 μg/m^3^ increase in PM_2.5_ concentration at lag01, stratified by age, sex, smoking status, alcohol consumption, hypertension, diabetes, and season. The *P* values indicate between-subgroup differences.

Figure 1 presents the subgroup-specific percent changes in HS hospitalization associated with each 10 μg/m^3^ increase in PM_2.5_ at lag01. Overall, positive associations were observed in most subgroups. The estimates were higher among patients aged ≥65 years and females, with percent changes of 3.80% (95% CI: 0.73, 6.97) and 3.50% (95% CI: 0.63, 6.46), respectively. Positive associations were also observed among patients with smoking and alcohol consumption, with corresponding estimates of 3.16% (95% CI: 0.37, 6.04) and 3.24% (95% CI: 0.44, 6.12). In addition, the estimates were relatively higher among patients with hypertension and diabetes, with percent changes of 4.54% (95% CI: 0.76, 8.47) and 4.08% (95% CI: 0.93, 7.33), respectively. By season, the association appeared stronger during the cold season, with a percent change of 5.26% (95% CI: 1.68, 8.96). However, no statistically significant differences were observed across subgroup comparisons.

## 4 Discussion

In this individual-level time-stratified case-crossover study, short-term PM_2.5_ exposure was associated with an increased risk of hospitalization for HS in Yancheng, China. The strongest association was observed within the current and previous day of exposure. The associations appeared more pronounced among females, older adults, patients with hypertension or diabetes, smokers, alcohol consumers, and during the cold season.

In the present study, each 10 μg/m^3^ increase in PM_2.5_ concentration was associated with a maximum 3.24% increase in the risk of HS hospitalization (95% CI: 0.55%−6.00%). Similar associations have been described previously, although the reported effect estimates differ substantially across populations and geographic regions. A case-crossover study conducted in Augsburg, Germany, found that each 10 μg/m^3^ increase in PM_2.5_ was associated with an approximately 11.5% increase in HS risk (95% CI: 2.90%−20.54%), which was notably higher than the effect observed in the present study (10). Likewise, the Women's Health Initiative in the United States suggested a positive association between long-term PM_2.5_ exposure and HS risk (9). Comparable findings were also reported in the United Kingdom, Portugal, and Poland, further supporting a link between air pollution exposure and HS (19–21). Studies from Asian countries have also reported positive associations between particulate matter exposure and hemorrhagic stroke. In Japan, each 10 μg/m^3^ increase in PM_2.5_ concentration was associated with an approximately 1%−2% increase in HS mortality (22), whereas the corresponding effect estimate in South Korea was approximately 7% for intracerebral hemorrhage (23). Within China, positive associations have also been reported across different regions. A time-stratified case-crossover study from Chengdu reported that each 10 μg/m^3^ increase in PM_2.5_ concentration was associated with an approximately 2.7% increase in intracerebral hemorrhage risk during 2014–2017 (24). Studies from Shanghai reported comparable associations, with overall effect estimates ranging from approximately 0.9% to 1.3%, while a stronger association was observed among individuals with hypertension, with an approximately 6% increase in risk (14, 25). Similar associations have also been reported in other Chinese regions, including Beijing and Shenzhen, while long-term exposure studies from Shandong further supported the association between PM_2.5_ exposure and hemorrhagic stroke outcomes (11, 13, 26). Nevertheless, not all studies yielded consistent findings. Nationwide analyses from Israel, Singapore, and Boston did not observe a clear positive association between PM_2.5_ exposure and HS (15, 18, 27). The effect estimate observed in our study was lower than those reported in several European and Korean studies, but was comparable to that observed in Chengdu and higher than estimates reported in some Shanghai studies. Such variations may partly reflect differences in population characteristics and susceptibility. The relatively high prevalence of hypertension in our study population (71.1%) may partly contribute to the higher effect estimate observed compared with some previous Chinese studies. Differences in regional air pollution environments, including variations in PM_2.5_ composition, pollution levels, climatic conditions, and emission sources, may also contribute to the heterogeneity in effect estimates. Additionally, the relatively low incidence of HS compared with ischemic stroke may introduce greater statistical uncertainty, resulting in greater variability in effect estimates across studies.

The effect of PM_2.5_ exposure on HS hospitalization in our study was mainly concentrated within the current and previous day of exposure, with the largest estimate observed for the 2-day moving average at lag01. No significant associations were identified at longer lag periods. However, previous studies have reported heterogeneous short-term lag patterns across different populations and exposure metrics. In Europe, the Augsburg study from Germany found that the effect of PM_2.5_ on HS was most evident at lag5–lag6, indicating a more delayed response pattern than that observed in our study (10). Similar delayed effects were also described in studies from the United Kingdom and Poland (19, 21). In contrast, studies from Asian populations tended to show earlier responses. In Japan, elevated PM_2.5_ concentrations on the previous day were associated with increased HS mortality, while studies from Shanghai, South Korea, and Chengdu reported that the associations were largely confined to the first few days following exposure (22–25). Similarly, a nationwide study involving 248 Chinese cities found that the association between PM_2.5_ and hemorrhagic stroke hospitalization varied across lag structures, with significant estimates observed at lag1 and lag2 but not at lag3, as well as across moving-average exposures from lag01 to lag03 (28). These findings suggest that the association between PM_2.5_ exposure and HS may be more pronounced during the early exposure period preceding the acute event. The stronger association observed at lag01 in our study may reflect the combined influence of PM_2.5_ exposure on the current and previous days, which represent the exposure period immediately preceding HS onset. By contrast, weaker associations observed with later exposure metrics may indicate attenuation of the short-term effect as the interval between exposure and HS onset increases. Such differences across studies may also reflect variations in study populations, pollutant characteristics, exposure assessment methods, outcome definitions, and statistical modeling strategies. Taken together, our findings appear more consistent with those reported in Asian populations, where the adverse effects of PM_2.5_ on HS may manifest relatively rapidly after exposure. Such a pattern may be biologically plausible, as previous studies have suggested that short-term PM_2.5_ exposure may affect endothelial function (29, 30), cerebral vascular regulation (29, 31), and autonomic activity (32–35), potentially contributing to increased susceptibility to acute cerebrovascular events shortly after exposure.

In the two-pollutant models, the association between PM_2.5_ exposure and HS hospitalization was attenuated after adjustment for SO_2_, NO_2_, and CO, whereas the associations at lag1 and lag01 remained statistically significant after adjustment for O_3_. This pattern suggests that the observed PM_2.5_ effects may partly reflect shared emission sources or collinearity with other traffic- and combustion-related pollutants. Similar findings have been reported previously. A multicity study from China demonstrated substantial attenuation of PM_2.5_-related stroke risk after controlling for NO_2_ or SO_2_ (15), while a Korean study likewise found weaker particulate matter effects in two-pollutant models, particularly after adjustment for traffic-related gaseous pollutants (23). In contrast, studies from the United States and Germany reported relatively stable associations after co-pollutant adjustment (9, 10). In our study, PM_2.5_ showed moderate-to-strong correlations with SO_2_, NO_2_, and CO (r = 0.40–0.74), but a weak correlation with O_3_ (*r* = −0.05). These correlation patterns may partly explain the greater attenuation observed after adjustment for SO_2_, NO_2_, and CO compared with adjustment for O_3_, as correlations among ambient pollutants may influence the estimated associations in co-pollutant models. Such differences across studies may partly arise from regional variations in pollutant mixtures, emission sources, and exposure characteristics (36).

Stratified analyses suggested stronger associations among females, older adults, individuals with hypertension or diabetes, smokers, alcohol consumers, and during the cold season, although no statistically significant differences were observed between subgroups. Similar patterns have also been described in previous studies (12, 18, 23, 24, 33). The larger effect estimates among females and older adults may reflect increased sensitivity to the vascular effects of PM_2.5_ exposure. Aging is frequently accompanied by impaired vascular function, reduced physiological reserve, and a greater burden of chronic diseases, which may predispose older individuals to acute cerebrovascular injury following particulate matter exposure (7, 33). In addition, sex-related differences in inflammatory responses, vascular regulation, and airway characteristics may partly explain the relatively greater effects observed among females (33, 34). Elevated effect estimates were also identified among patients with hypertension and diabetes. PM_2.5_ exposure may further increase blood pressure and vascular instability in hypertensive individuals, thereby increasing the likelihood of cerebral vessel rupture (37, 38). Meanwhile, both diabetes and particulate matter exposure are associated with systemic inflammation, endothelial dysfunction, and vascular injury, which may together contribute to a heightened risk of HS (34, 35, 39). Stronger associations were likewise observed among smokers and alcohol consumers. Smoking-related airway irritation and cough may transiently elevate intracranial pressure through a Valsalva-like effect, potentially promoting rupture of vulnerable cerebral vessels or aneurysms (10), while alcohol consumption may contribute to blood pressure fluctuations, impaired vascular regulation, and increased cerebrovascular fragility (40). In addition, the more pronounced associations during the cold season are consistent with findings from previous studies conducted in China and other Asian regions (12, 24), and may partly reflect higher pollution concentrations, reduced atmospheric dispersion, and enhanced vasoconstrictive responses during colder weather. Overall, these findings suggest that the cerebrovascular effects of short-term PM_2.5_ exposure may be amplified in populations with greater physiological vulnerability.

Several limitations should be acknowledged. First, exposure assessment was based on fixed-site ambient monitoring data rather than individual-level measurements. Although this approach is widely used in time-stratified case-crossover studies of ambient air pollution, it may not fully capture individual variations in exposure arising from residential location, indoor environments, occupational activities, commuting patterns, and other daily behaviors, potentially leading to exposure misclassification. Second, although the time-stratified case-crossover design controls for stable individual characteristics, residual confounding from time-varying factors, such as indoor air pollution, physical activity, medication use, occupational exposure, and short-term behavioral changes, could not be completely excluded. Moreover, although two-pollutant models were conducted as sensitivity analyses to assess the influence of co-pollutant adjustment on the observed association between PM_2.5_ exposure and HS risk, multi-pollutant models were not performed because of the limited number of HS events and potential correlations among ambient pollutants, which may increase uncertainty in effect estimates. Third, because the hospitalization records were anonymized, longitudinal follow-up information at the individual level was unavailable. Therefore, the exact timing of symptom onset and the longitudinal history of previous HS events could not be fully captured. Consequently, the hospitalization date was used as the event date, and first-ever HS events could not be distinguished from recurrent HS events, limiting further evaluation of whether the associations differed between these two types of events. Fourth, this study was conducted in a single city in eastern China, and the findings may not be fully generalizable to regions with different pollution sources, climatic conditions, or population characteristics. Fifth, although several biological pathways, including endothelial function, cerebral vascular regulation, and autonomic activity, have been proposed to explain the association between PM_2.5_ exposure and hemorrhagic stroke, relevant biological markers were not measured in the present study. Future studies incorporating biological measurements are needed to further elucidate the underlying mechanisms. In addition, PM_2.5_ was evaluated primarily on the basis of mass concentration, while detailed information on chemical constituents and source-specific components was unavailable. Future studies incorporating particle composition and source apportionment may help clarify the specific toxic fractions most strongly associated with HS risk.

## 5 Conclusions

In conclusion, this individual-level time-stratified case-crossover study suggests that short-term PM_2.5_ exposure is associated with an increased risk of HS hospitalization in Yancheng, China. These findings may have important implications for the prevention of HS associated with short-term PM_2.5_ exposure, particularly among susceptible populations.

## Data Availability

The raw data supporting the conclusions of this article will be made available by the authors, without undue reservation.
